# A great simulator in clinical practice: mononeuritis multiplex in HIV infection

**DOI:** 10.4322/acr.2024.493

**Published:** 2024-06-14

**Authors:** José Pedro Soares Baima, Beatriz Carneiro Gondim Silva, Vanessa Lopes Vieira, Luiz Pedro Meireles, Milton Hideaki Arai, Angelina Maria Martins Lino

**Affiliations:** 1 Universidade de São Paulo (USP), Hospital das Clínicas, Faculdade de Medicina, Departamento de Neurologia, São Paulo, SP, Brasil; 2 Universidade de São Paulo (USP), Hospital das Clínicas, Faculdade de Medicina, Departamento de Medicina Interna, São Paulo, SP, Brasil

**Keywords:** Biopsy, HIV, Mononeuropathies, Vasculitis

## Abstract

HIV infection is a chameleon, mimicking several diseases. Herein, we report a previously healthy 39-year-old woman who, over 2 months, developed arthritis, weight loss, and confluent multiple mononeuropathy. Extensive laboratory investigation showed positive serology for HIV, with a CD4 count of 100 cells, and necrotizing vasculitis on a nerve biopsy not associated with CMV co-infection, allowing the diagnosis of polyarteritis nodosa-like vasculitis in an HIV-infected patient. Apart from the infection, HIV-related autoimmunity can affect any organ and contribute to the complexity of the clinical presentation of HIV infection.

## INTRODUCTION

Infectious neuropathy is a joint worldwide group of entities of difficult diagnosis in non-endemic areas.^1^ HIV is still a burden that seems far from an end, with alarming incidence rates in low-income countries.^2^ In 2021, 960,000 people were living with HIV infection in Brazil.^3^ The HIV infection involving the central and peripheral nervous system may mimic several neurological and non-neurological diseases. Chronic distal symmetric sensory neuropathy affects one-third of patients with HIV infection, even in treated patients with newer and less neurotoxic antiretroviral drugs.^4^ Autoimmunity linked to HIV infection is a known process, which can be unrecognized.^5^ Among HIV-related autoimmunity, a series of small, medium, and even large vessel vasculitis has been described, with a frequency lower than 1%.^6^ Vasculitis is a challenging diagnosis in everyday practice, even when presenting as its most common form. We herein present a case of polyarteritis nodosa-like in an unknown HIV infection.

## CASE REPORT

**Figure 1:**
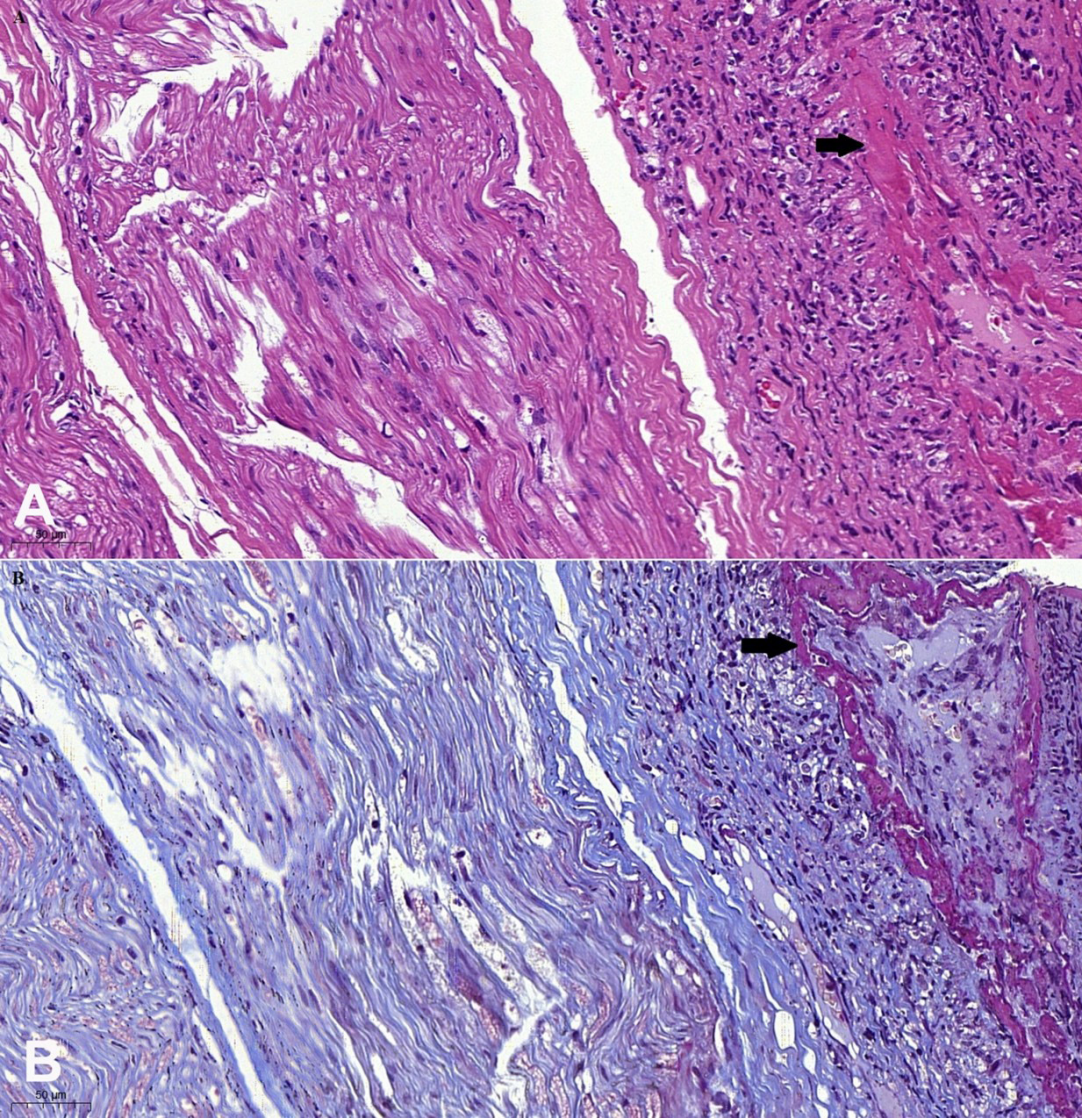
Photomicrograph of the sural nerve showing fibrinoid necrosis (arrows) in epineural arteries on longitudinal sections in **A –** (H&E) and in **B –** (Trichrome stain). Bar of 50 micra.

**Figure 2:**
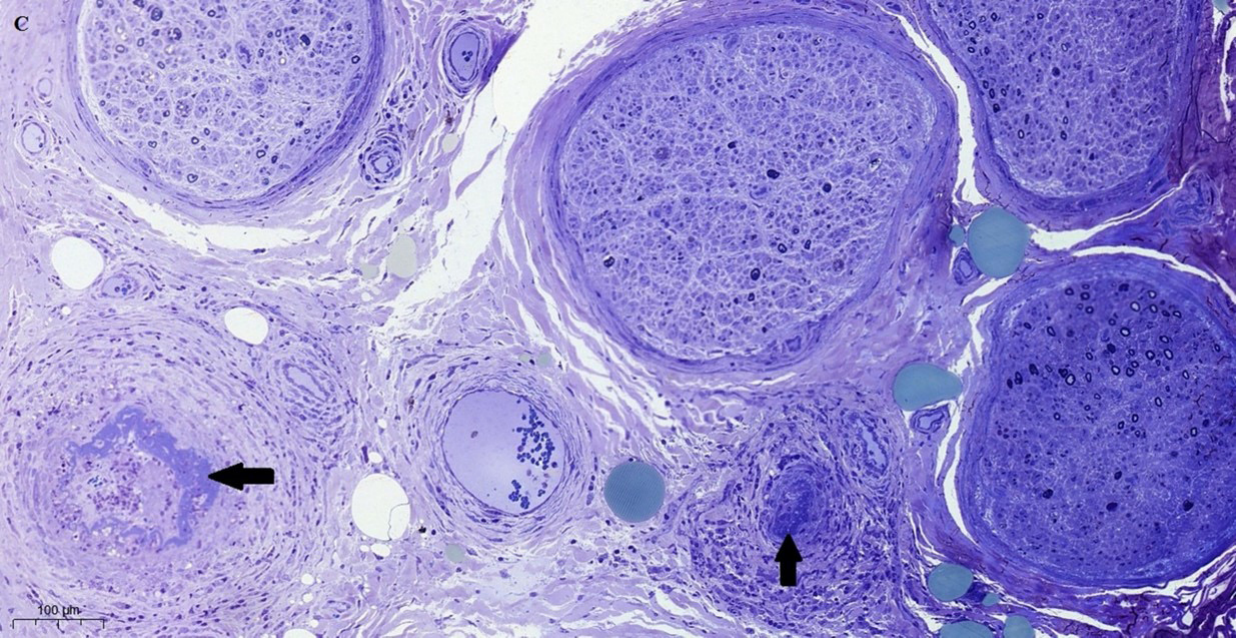
Photomicrograph of the sural nerve. Semi-thin transverse section with asymmetrical loss of nerve fibers between nerve fascicles and fibrinoid necrosis (arrows) - (Toluidine blue). Bar of 100 micra.

A 39-year-old woman sought medical care with a 2-month history of arthritis, fever, and weight loss. She complained of asymmetric pain in the lower limbs with progressive weakness leading to the inability to walk. Shortly before her hospital admission, she complained of tunnel vision in the left eye and transient color blindness with a spontaneous recovery in the right eye. Her physical examination was remarkable for paraparesis, with a grade 0 on feet dorsiflexion and plantar flexion, grade II on legs extension, and a grade V on upper limbs. Deep tendon reflexes were normal in the upper limbs and absent in the lower limbs. Cranial nerves examination revealed only blindness in lower quadrants (altitudinal blindness) on confrontation visual fields, highly suggestive of ischemic optic neuritis. Electrodiagnostic studies disclosed severe distal sensory-motor axonal polyneuropathy in lower limbs (Table 1). The distal and slightly lower limbs involvement with normal findings in the upper limbs, systemic symptoms, and the electrodiagnostic studies raised the concern of a confluent mononeuritis multiplex. Laboratory studies disclosed the HIV infection (90,600 copies/mL) with a CD4 count of 100 cells/mm^3^ (reference range [RR]; 500 – 1500 cells/mm^3^). Lumbar cerebrospinal fluid examination (CSF) showed 26 cells/mm^3^ with a lymphocytic predominance (86%), protein of 89 mg/dL (RR; 40 – 45 mg/dL), and increased gamma globulins fractions on electrophoresis. Extensive autoimmune panel was negative, except for a lupus anticoagulant of 1.29 (RR < 1.16), which was repeated after 12 weeks with negative results, but the patient was already on corticosteroid treatment. A sural nerve biopsy showed arteritis with fibrinoid necrosis (Figures 1 and 2).

**Table 1:** Electrodiagnosis studies

**Motor Studies**						
	**Right**			**Left**		
**Nerve**	**Lat**	**Amp (mV)**	**CV (m/s)**	**Lat**	**Amp (mV)**	**CV (m/s)**
Median	3.5	13	54.7	3.1	11.9	54.7
Ulnar	2.7	17.5	61	2.5	15.7	60.8
Tibial	5.5	0.4	34.1	4.8	0.2	36
Fibular	U	U	U	4.2	0.1	39.5
**Sensory Studies**						
	**Right**			**Left**		
**Nerve**	**Lat**	**Amp (μV)**	**CV (m/s)**	**Lat**	**Amp (μV)**	**CV (m/s)**
Median	3.5	25.4	52.2	3.2	38.3	56.9
Ulnar	3	21.1	49.6	2.9	36.4	57.1
Radial	2.2	28.6	60.2	1.9	37	71.4
Superficial fibular	U	U	U	U	U	U
Sural	U	U	U	U	U	U
**H-Reflex**	**Right**	**Left**				
Tibial nerve	33	32.8				

Amp – amplitude; CV – conduction velocity; Lat: latency; mV – millivolts; μV– microvolts; m/s – meters per second; U – unexcitable nerve.

Immuno-histochemistry for cytomegalovirus was negative on sural nerve biopsy. She was diagnosed with polyarteritis nodosa-like related to HIV infection. She was treated monthly with intravenous methylprednisolone (1000 mg/day for 03 days for six months). After intravenous corticosteroid, she regained her walking ability. During the follow-up, the patient had a mild neurological recurrence and arthritis in the left foot during highly effective antiretroviral therapy, but it was successfully managed with oral corticosteroid adjustment.

## DISCUSSION

The most common presentation of neuropathy in HIV patients is chronic distal symmetric sensory neuropathy. Initially attributed to uncontrolled viremia and drug toxicity, even during highly active antiretroviral therapy with lesser neurotoxicity, up to 30% of patients present chronic sensory neuropathy. This pattern is associated with mild loss in the sensory examination with absent tendinous reflexes in a symmetric length-dependent distribution. Prominent weakness is not usually associated and should concern other neuropathy types.^4^ Although chronic symmetric sensory neuropathy can be painful and distressful, it develops over a long period with gradual onset. In our patient, the onset of the neuropathy was acute, with painful symptoms and prominent weakness, leading to the inability to walk. Other clues were asymmetry in neuropathic involvement with only distal involvement and concomitant systemic symptoms, a pattern that raised suspicion of a confluent mononeuritis multiplex. Facing this pattern, vasculitis should always be considered.

A pitfall in clinical practice is confluent mononeuritis, which can be only slightly asymmetric or mimic length-dependent symmetric neuropathy, called polyneuropathy.^7^ As a classic example of confluent mononeuropathy, our patient had an asynchronous symptoms onset, which is sufficient to hypothesize mononeuritis multiplex. Symmetrical physical examination can be misleading. Neurophysiological studies most often identify an asymmetrical involvement. When electrodiagnostic studies are not enlightening, an important diagnostic clue is a history of stepwise clinical involvement, in which individual nerve deficits are summated over time. Usually, vasculitis has a lower limb predominance, sensory-motor symptoms with acute to subacute features, and is often painful. Peroneal nerves are damaged in more than 90% of peripheral nervous system vasculitis, as observed in the absence of dorsiflexion in our patient.^8^

Acute painless visual loss should always be a concern since it could be associated with permanent blindness. Our patient confrontation visual field test revealed an altitudinal visual abnormality, typical for anterior ischemic optic neuritis (AION). This condition is divided into arteritic and non-arteritic AION. Besides altitudinal visual blindness, fundoscopy is remarkable for a swollen disc in both AION types. In arteritic AION, the leading cause is temporal arteritis, which affects individuals aged between 70-75 years, usually accompanied by other systemic symptoms that do not fit well with our patient.^9^

A reasonable question is whether HIV is associated with autoimmunity and its role in vasculitis. HIV depresses the immune system, including regulatory T cells. The disbalance in CD4/CD8 counts enhances the cytotoxicity, contributing to antigen exposures. Not only is T-CD8+ hyperactivated, but B lymphocytes are hyperactive, producing antibodies. The catabolism and weight loss could intensify the antigens exposure, triggering autoimmunity due to impaired autophagy with the accumulation of cell debris and senescent cells, maximizing antigen exposure in a dysregulated immune system. One should not disregard molecular mimicry as a potential cause of autoimmunity in HIV infection, as frequently credited in other diseases.^10,11^

Polyarteritis nodosa (PAN) predominantly affects middle-sized vessels and is usually associated with the hepatitis B virus but is possibly the most common HIV-associated vasculitis. Only a few clinical differences between classical and HIV-related polyarteritis nodosa are reported in the literature. In HIV, the clinical course is more acute than the classical PAN form, the kidney is less frequently involved, and a better response to corticosteroid treatment is observed.^12^ Other viruses implicated in viral-associated polyarteritis nodosa are Hepatitis-C and Parvovirus B19.^7^

An extensive workup should always be performed in cases of multiple mononeuropathy. Seropositivity for autoantibodies needs to be checked regularly, especially ANCA antibodies, which are paramount in vasculitic neuropathies. Organ dysfunction should constantly be screened, even when there are no clear signs or symptoms of systemic involvement. Electrodiagnostic studies are helpful in checking for an expected axonal injury pattern, asymmetric nerve involvements, and types of fiber loss, and, more importantly, disclose nerve subclinical involvement. Pseudo-conduction block is evidence of nerve infarction and could be mistakenly interpreted as a conduction block due to a demyelinating lesion. Pseudo-conduction blocks represent local ischemia that disrupts the action potential and generates focal conduction failure. If a new nerve conduction study is carried out after a few weeks, signs of axonal damage appear due to Wallerian degeneration.^7^

Nerve biopsy is the standard diagnostic procedure for investigating a possible autoimmune vasculitis presenting as a mononeuritis multiplex, especially when non-nervous system biopsy sites are unavailable.^13^ When affected, the sural nerve is the most frequently chosen, although it is not entirely harmless. Lateral foot hypoesthesia is expected after the procedure and can be an everlasting sequel in addition to the rare local neuropathic pain associated with neuroma formation in the proximal stump. If significant asymmetry between sural nerves is identified, the nerve with the worst sensory action potential amplitude is chosen to minimize numbness after the procedure.

Pathologic classification of peripheral nerve vasculitis involves definite, probable, and possible vasculitis criteria. In our patient, inflammatory infiltrate and fibrinoid necrosis in the vascular wall fulfill the Level 1 case definition established by the Brighton Collaboration Vasculitic Neuropathy Working Group.^14^ The core requirement for definite vasculitic neuropathy is intramural inflammation with evidence of necrosis of the vascular wall. Other categories encompass asymmetric interfascicular or intrafascicular axonal loss and vessel wall damage without fulfilling the definite pathological criteria.^15^

A differential diagnosis of utmost importance is the presence of opportunistic infections, especially in patients without treatment. Cytomegalovirus (CMV) infection should be the main concern and be searched by tissue immunohistochemistry.^12^ No evidence of CMV was found on the sural nerve biopsy of our patient. In patients with meager CD4 cell count (< 50 cells), CMV can present as an acute lumbosacral radiculitis with an acute proximal asymmetrical weakness and could evolve to cauda equina syndrome. That was not the case with our patient since no sphincter abnormalities were present, and distal rather than proximal involvement was observed on physical examination.

Finally, two presentations of HIV neuropathy are noteworthy: acute inflammatory demyelinating polyradiculopathy (AIDP) and chronic inflammatory demyelinating neuropathy (CIDP). HIV-associated acute inflammatory demyelinating polyradiculopathy is virtually indistinguishable from the classic idiopathic Guillain-Barré syndrome, even in complementary exams like spinal fluid and electroneuromyography. According to the literature, in both AIDP and CIDP, a hint of secondary or associated etiology is pleocytosis of 50 cells/mm^3^ or more in spinal fluid cytology; however, in our clinical practice, the suspicion arises with cellularity greater than 10 cells/mm^3^.^4^ HIV should be part of the screening in every patient with chronic inflammatory demyelinating polyradiculopathy since it is a known cause of associated CIDP and is not different from idiopathic CIDP.^16^ These two forms of HIV-associated neuropathy differ from our patient’s clinical presentation in its temporal course, asymmetric onset, and axonal pattern in electrophysiological studies.

## CONCLUSIONS

HIV infection has a wide range of presentations and could be a challenge to internal medicine physician and neurologists in the care of patients. HIV should be sought in patients presenting with mononeuritis multiplex, presenting in any age group. Not only direct virus-associated neuropathy but also opportunistic infections should be a concern in HIV patients. Knowledge of types of peripheral nerve involvement in HIV and steps of clinical reasoning, as demonstrated in this paper, can lead physicians to a better and faster diagnosis. Tissue biopsy remains the standard for diagnostic confirmation in most vasculitis cases.^17^ Unfortunately, nerve biopsy is not readily available in Brazil. In patients with polyarteritis-nodosa-like in HIV infection, corticosteroid response is good with an excellent prognosis, as in the case presented herein.
